# Achene micromorphology and its taxonomic significance in some species in *Taraxacum* sect. *Palustria* (Asteraceae)

**DOI:** 10.3897/phytokeys.166.54271

**Published:** 2020-10-29

**Authors:** Agnieszka Rewicz, Jolanta Marciniuk, Paweł Marciniuk

**Affiliations:** 1 University of Lodz, Department of Geobotany and Plant Ecology, 12/16 Banacha, 90-237 Lodz, Poland University of Lodz Lodz Poland; 2 Siedlce University of Natural Sciences and Humanities, Faculty of Exact and Natural Sciences, 14 B Prusa, 08-110 Siedlce, Poland Siedlce University of Natural Sciences and Humanities Siedlce Poland

**Keywords:** achene, micromorphology, scanning electron microscopy (SEM), *
Taraxacum
*, taxonomy

## Abstract

The genus *Taraxacum* is one of the largest and taxonomically most complicated apomictic genera. Currently, it is considered to consist of over 2800 species placed within 60 sections. Due to the large number of species, and their uniform morphological design and plasticity of leaves, the identification of plants at the species level is challenging even for specialists. This problem significantly hinders the study of their properties and the rational use of these valuable medicinal and nutritional plants. This paper presents the results of research on the morphology and micromorphology of achenes of 28 Taraxacum species of sect. Palustria and for comparison one species per section of: *Erythrosperma*, *Naevosa*, *Piesis*, and *Taraxacum*. The achenes were measured with a stereoscopic microscope and a biometric program, and micromorphological studies were performed by scanning electron microscopy. It has been shown that traits associated with achene morphology and micromorphology have a high diagnostic value, allowing us to distinguish sections as well as species within the sect. Palustria. Based on the examined achene features, a dichotomous key for determining the studied species was constructed.

## Introduction

The family Asteraceae is a large family of flowering plants with over 25,000 species and 1620 genera (Stevens 2001). Asteraceae species can be found in all continental zones and they play a significant role in phytocenoses (Takhtajan 2009). *Taraxacum* is one of the largest and most taxonomically complex apomictic genera. Its wide variety of forms is reflected in over 2,800 species described so far, clustered in about 60 sections (Kirschner et al. 2015; Kirschner and Štěpánek 1997, 2004, 2008; Uhlemann et al. 2004). Among its main mechanisms, Kirschner and Štěpánek (1996) include multiple hybridization, polyploidization, and apomixis that fix hybrids with odd ploidy levels. At high latitudes of the Northern Hemisphere, where sexually reproducing species are scarce, the emergence of new forms (microevolution) is caused by autopolyploidization processes and somatic mutations perpetuated by apomixis (Kirschner and Štěpánek 1996; Mes et al. 2002; Majeský et al. 2012). The genetic variability is superimposed by the very high phenotypic plasticity of dandelions, which significantly increases the already serious problems with species identification (Kirschner et al. 2016). Phenotypic plasticity of the species is a form of adaptation of the plant to habitat conditions and an expression of its potential for colonizing areas that differ in many habitat features (Stace 1993; Sultan 1995, 2000, 2001). The features that are the least susceptible to environmental changes, and thus used in taxonomy, are the features of flower and seed, fruit, and leaf arrangement (Heywood 1967). On the other hand, the following features are the most susceptible to environmental changes: shoot height, leaf size and color, flower size and flowering time (Heywood 1967; Stace 1993; Sultan 1995).

The identification problem resulting from high variability, among others, also applies to the relatively thoroughly investigated *Taraxacum* sections, including one of the most vulnerable sections ‒ *Palustria* ‒ to which this study is devoted.

Taraxacum sect. Palustria has about 160 described and confirmed species (Hudziok 1969; Kirschner and Štěpánek 1998; Štěpánek and Kirschner 2001, 2017; Tikhomirov 2003; Aquaro et al. 2008; Carlesi and Peruzzi 2012; Marciniuk et al. 2012; Marciniuk et al. 2018). They are almost exclusively apomictic polyploids, from triploids (2n = 24) to hexaploids (2n = 48). Sexual diploids are represented only by the two southern European species *Taraxacum raii* and *T. tenuifolium*. In the Palustria section monograph of Kirschner and Štěpánek (1998), they developed a multi-access key for species identification which was based mainly on the morphology of flowers and inflorescences. In practice, this key allows for the determination of only very typical specimens with a set of well-developed diagnostic features for a species (or only a group of species). Such plants are rare in nature, and searching for them requires a great deal of knowledge and experience (Kirschner et al. 2016). Atypical specimens, challenging to identify, predominate in herbarium materials, which often leads to erroneous determinations. Such misidentifications have serious consequences, especially where *Taraxacum* is used for medical and cosmetic purposes. It is therefore essential to find new features and patterns to help identify dandelions.

One technique that allows for the determination of new diagnostic features is SEM (scanning electron microscopy). SEM had already been used in the first half of the 20^th^ century (Zworykin et al. 1942). Since that time, SEM has been used for the identification of species in different groups of organisms such as bacteria, fungi, lichens, mosses, and vascular plants (Duckett and Soni 1972; Jamjoom 2007; Rewicz et al. 2017a, b; Fass 1973; Ullah et al. 2018). Nowadays, SEM is playing a useful role in taxonomic research of plants. It has been used to describe the ultrastructure of, e.g., fruits and seeds (Ullah et al. 2019a, b; Hadidchi et al. 2020). Many studies have shown that fruit or seed can very well serve in the identification, classification, and delimitation of species at various taxonomic levels (Clifford and Smith 1969; Bacchetta et al. 2011; Gamarra et al., 2008, 2010; Heneidak and Khalik 2015; Martín-Gómez et al. 2019a, b). Moreover, biometric seed and fruit sculpture analysis has proved to be a useful tool for phylogenetic inference (Saadaoui et al. 2013).

The application of SEM for micromorphologic evaluation in taxonomic research in the family Asteraceae seems to be promising (Kreitschitz and Vallès 2007; Bona 2015; Karaismailoglu 2015; Ozcan and Akinci 2019). Unfortunately, in the case of the genus *Taraxacum*, despite its size and taxonomic complexity, such research is scarce to date. The exceptions are the works of Savadkoohi et al. (2012) concerning 17 species from different sections; Dudáš et al. (2013) concerning four species from the section Erythrosperma; Marciniuk et al. (2009), in which achenes *T. scanicum* and *T. bellicum* (under the name *T. prunicolor*) were compared; Wu et al. (2011) concerning 10 species from North-East China; and a compilation of scanning microscopic images of achenes of selected species from Taraxacum sect. Palustria and T. sect. Erythrocarpa (Schmid 2002; Bednorz and Maciejewska-Rutkowska 2010; Marciniuk 2012), but without in-depth comparative studies.

It is important to develop a functional key for the genus *Taraxacum*, based on the largest possible number of features, including relatively stable fruit properties. Features associated with the morphology of leaves and inflorescences are highly variable (Kirschner et al. 2016), and usually poorly developed in the case of marsh dandelions not growing in optimal habitats. The determination of such plants is challenging and connected with a high risk of errors. Dandelions are essential medicinal and food plants as well as model organisms, among others, in research on apomixis. Thus, the high probability of erroneous determinations or the use of ambiguous collective names in publications reduces the credibility of the study and may even lead to undermining it (Kirschner et al. 2016). In our research, we focused primarily on the Central European section Palustria, whose representatives were compared with selected species from four sections (*Erythrosperma*, *Piesis*, *Taraxacum*, and *Naevosa*) with which Taraxacum sect. Palustria could have been crossed in the past.

The presented study aims to use micromorphological characters (based on scanning electron microscopy and biometrical traits) of achenes for the taxonomic identification and species delimitation in some species of the genus *Taraxacum*.

## Materials and methods

### Biometric analyses of achene body

**Figure 1.**
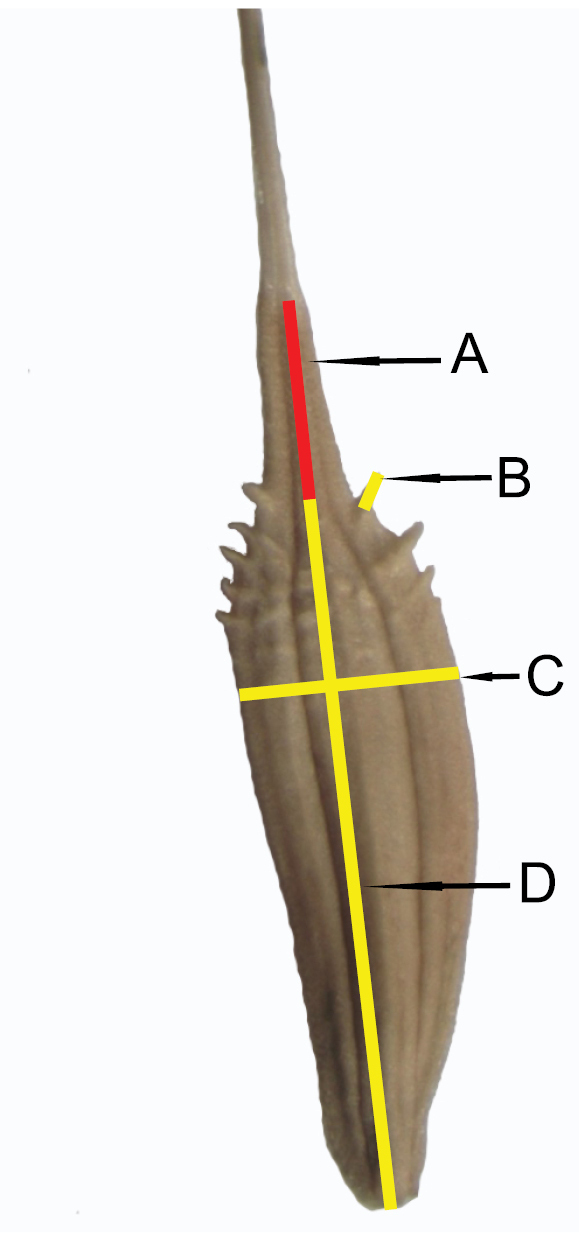
Measurement scheme of achenes: **A** cone length **B** spinule length **C** achene width **D** achene length.

We analyzed achenes from 28 Taraxacum species, 24 of which belong to sect. Palustria and 4 species to four other sections included for comparison: *Taraxacum bessarabicum* of sect. Piesis, *T. bellicum* (in the sense of the description of *T. prunicolor* by Schmid et al. 2004) of sect. Erythrosperma, *T. gelertii* of sect. Naevosa, and *T. linearisquameum* of sect. Taraxacum. Marsh dandelion species have been chosen to represent the majority of Central European species groups (according to Kirschner and Štěpánek 1998). Achenes were collected from properly developed plants from natural habitats (Table 1) for each species. From ten plants, at least 30 correctly developed and undamaged achenes were randomly selected for measurements. Herbarium sheets were deposited in the Herbarium of Siedlce University of Natural Sciences and Humanities (WSRP), Poland. Five achene traits were quantified: A) cone length, B) spinule length, C) achene width, D) achene length (without cone) (Fig. 1), and E) index ‒ the ratio of achene length to cone length. The achene traits were measured automatically in the horizontal view using the biometric program OptaView-IS version 4.3.0.6001. The observations and measurements of the achene were performed with the use of OPTA-TECH optical stereomicroscope (Table 1). To analyze ornamentation on the achene body, we took about 50 measurements of spinules from the surface of achenes (three to five individuals from each species). Measurements were done based on SEM images.

**Table 1.** List of studied species of *Taraxacum* included in this study. N –latitude, E – longitude; abbreviations: the first capital letter from the genus and the three first letters from the species name; bold: species from outgroup sections.

Species	Abbreviation	Number of analyzed achenes	Locality	N / E	Habitat
*T. ancoriferum* Hudziok in Feddes Repert. 80: 333. 1969	*T. anc*	43	Czuchów	52°17', 22°44'	wet meadow
*T. balticum* Dahlst. in Bot. Not. 1905: 162. 1905	*T. bal*	38	Pyzdry	52°09', 17°41'	salt meadow
*T. bavaricum* Soest in Acta Bot. Neerl. 14: 21. 1965	*T. bav*	42	Czuchów	52°17', 22°44'	wet meadow
***T. bellicum*** (sect. Erythrosperma) Sonck in Memoranda Soc. Fauna Fl. Fenn. 59: 1. 1983	*T. bel*	51	Nowogród	53°13', 21°52'	psammophilous grassland
*T. belorussicum* Val. N. Tikhom. in Novosti Sist. Vysš. Rast. 35: 207. 2003	*T. belo*	39	Mścichy	53°25', 22°30'	peat bogs in the Biebrza Valley
***T. bessarabicum*** (sect. Piesis) (Hornem.) Hand.-Mazz. in Monogr. Taraxacum: 26. 1907	*T. bes*	42	Košice	48°45', 21°15'	city lawn
*T. brandenburgicum* Hudziok in Feddes Repert. 75: 131. 1967	*T. bra*	48	Pyzdry	52°09', 17°41'	salt meadow
*T. dentatum* Kirschner & Štěpánek in Thaiszia 4: 156. 1994	*T. den*	50	Czuchów	52°17', 22°44'	wet meadow
*T. fascinans* Kirschner, Mikoláš & Štěpánek in Preslia 69: 45. 1997	*T. fas*	42	Bydgoszcz	53°06', 18°07'	wet meadow
***T. gelertii*** (sect. Naevosa) M. P. Christ. in Rosenvinge & Warming, Bot. Iceland 3: 303. 1942	*T. gel*	51	Władysławowo	54°47', 18°24'	wet meadow
*T. hollandicum* Soest in Ned. Kruidk. Arch. 52: 226. 1942	*T. hol*	47	Kozłówek	49°50', 21°40'	wet meadow
***T. linearisquameum (set. Taraxacum)*** Soest in Proc. Kon. Ned. Akad. Wetensch., Ser. C, Biol. Med. Sci. 69: 471. 1966	*T. lin*	31	Lipnica Wielka	49°42', 20°43'	city lawn
*T. madidum* Kirschner & Štěpánek in Thaiszia 4: 149. 1994	*T. mad*	30	Krościenko	49°25', 20°25'	calcareous fen
*T. mariae* J. Marciniuk & P. Marciniuk in Phytotaxa 376 (5): 208. 2018	*T. mar*	33	Modliborzyce	50°45', 22°19'	wet meadow
*T. mendax* Kirschner & Štěpánek in Folia Geobot. Phytotax. 20: 413. 1985	*T. men*	56	Matulnik	49°52', 22°06'	wet meadow
*T. paucilobum* Hudziok in Feddes Repert. 72: 29. 1965	*T. pau*	41	Krześlin	52°13', 22°21'	wet meadow
*T. pauckertianum* Hudziok in Feddes Repert. 80: 328. 1969	*T. pauc*	38	Polanowo	52°24', 17°55'	wet meadow
*T. polonicum* Małecka & Soest in Acta Biol. Cracov., Ser. Bot. 15: 119. 1972	*T. pol*	48	Wiślica	50°21', 20°40'	wet meadow
*T. portentosum* Kirschner & Štěpánek in Monogr. Taraxacum Sect. Palustria: 233. 1998	*T. por*	32	Krześlin	52°13', 22°21'	wet meadow
*T. skalinskanum* Małecka & Soest in Acta Biol. Cracov., Ser. Bot. 15: 120. 1972	*T. ska*	52	Modlniczka	50°07', 19°50'	wet meadow
*T. subalpinum* Hudziok in Feddes Repert. 72: 26. 1965	*T. sub*	38	Pyzdry	52°09', 17°41'	wet meadow
*T. subdolum* Kirschner & Štěpánek in Preslia 64: 28. 1992	*T. subd*	41	Czuchów	52°17', 22°44'	wet meadow
*T. subpolonicum* Kirschner & Štěpánek in Monogr. Taraxacum Sect. Palustria: 120. 1998	*T. subp*	34	Wilczonek	52°10', 22°02'	wet meadow
*T. telmatophilum* Kirschner & Štěpánek in Preslia 58: 104. 1986	*T. tel*	51	Gotówka	50°10', 23°32'	calcareous fen
*T. trilobifolium* Hudziok in Feddes Repert. 75: 134. 1967	*T. tri*	46	Krześlin	52°13', 22°21'	wet meadow
*T. udum* Jord. in Mém. Acad. Roy. Sci. Lyon, Sect. Lett., ser. 2 1: 325. 1851	*T udu*	42	Pomiechówek	52°27', 20°43'	wet meadow
*T. vindobonense* Soest in Acta Bot. Neerl. 14: 50. 1965	*T. vin*	48	Krześlin	52°09', 17°41'	wet meadow
*T. zajacii* J. Marciniuk & P. Marciniuk in Ann. Bot. Fenn. 49: 388. 2012	*T. zaj*	53	Harta	49°51', 22°13'	wet meadow

The length of spinules was chosen as the criterion for dividing the analyzed species into three types:

Type A) very short spinules with a free part length of 5 to 10 microns;

Type B) medium spinules with a free part length from 11 to 19 microns;

Type C) very long spinules with a free part length of 20 to 30 microns.

The terminology of ornamentation of achene body is based on Stearn (2004).

### SEM analyses

Micromorphological data were obtained by SEM (Phenom Pro X) at the Department of Invertebrate Zoology and Hydrobiology, University of Lodz (Poland). The achenes were sputter-coated with a 4 nm layer of gold. The achene surface ultrastructure 3D models were made using 3D Roughness Reconstruction software from the Phenom Suite. The digital images obtained by SEM were trimmed and arranged in plates using Corel Draw 2018.

### Statistical analyses

The following basic characteristic features were calculated: arithmetic average (x), maximum and minimum values (max and min), standard deviation (SD), and coefficient of variation (CV). A cluster analysis (CA) on the shortest Euclidean distances according to Ward’s method was applied to determine the number of clusters between taxa. To differentiate between species, a K-means clustering analysis was conducted. The optimal number of K-groups was determined based on the results of agglomeration analysis.

The Shapiro-Wilk and Kolmogorov-Smirnov tests were conducted to check for a normal distribution of the data; both were not normal, and therefore, the Kruskal-Wallis test (for *P* ≤0.05) was used, as a nonparametric alternative to ANOVA (Zar 1984).

The software packages STATISTICA PL. ver. 13.1 and MVSP 4.5 were used for all the mentioned numerical analyses (van Emden 2008).

## Results

### Biometrical studies of achene body

**Figure 2.**
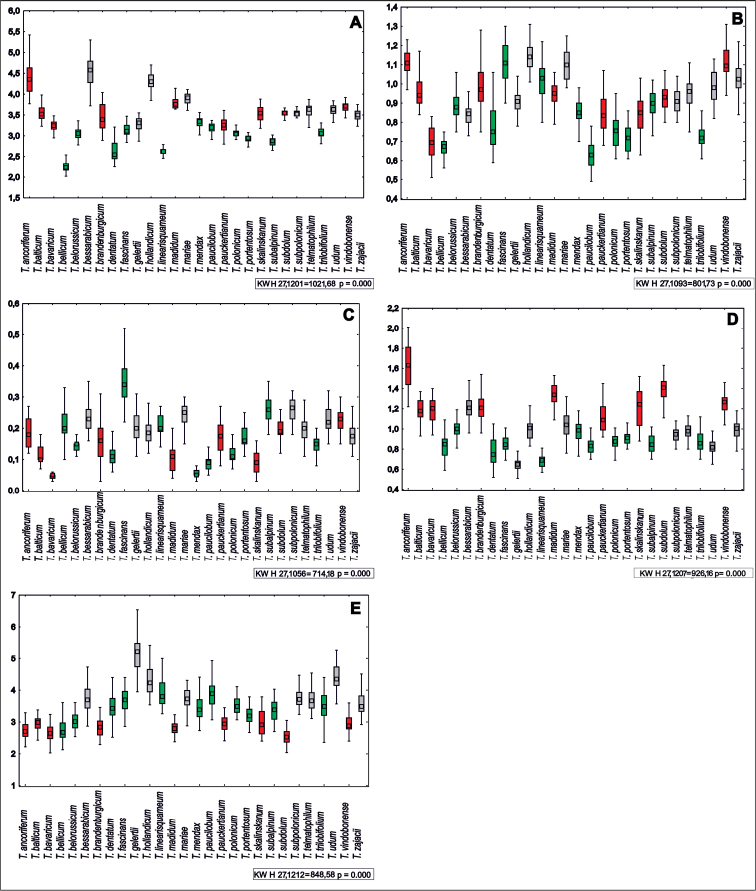
**A–E** Ranges of variation of traits of *Taraxacum* taxa: boxes represent the 25^th^–75^th^ percentiles, the upper and lower whiskers extend the minimum and maximum data point, the square inside the box indicates median, while colors indicate K-groups 1: gray, 2: red, 3: green, **A** achene length **B** achene width **C** spinule length **D** cone length **E** index.

The analysis of achene traits revealed various informative features useful for the identification of species within this section. The longest achenes occur in *T. ancoriferum* (4.42 mm) and *T. hollandicum* (4.30 mm), while the shortest achenes occur in *T. bellicum* (sect. Erythrosperma), *T. linearisquameum* (sect. Taraxacum), *T. subalpinum*, and *T. portentosum* (2.34 mm, 2.63 mm, 2.86 mm, and 2.93 mm, respectively). The widest achenes occur in *T. hollandicum* (1.14 mm), *T. ancoriferum* (1.11 mm), and *T. fascinans* (1.11 mm). The narrowest achenes were recorded for the following species: *T. paucilobum* (0.62 mm), *T. bellicum* (0.68 mm), and *T. bavaricum* (0.69 mm). The species with the longest cones were *T. ancoriferum* (1.63 mm), *T. subdolum* (1.39 mm), and *T. madidum* (1.34 mm). The smallest cones were found in *T. gelertii* (sect. Naevosa) and *T. linearisquameum* (0.65 mm and 0.68 mm, respectively). The highest index occurs in *T. gelertii* (5.14), *T. udum* (4.46), and *T. hollandicum* (4.34), while the shortest index occurs in *T. ancoriferum* (2.70) and *T. bavaricum* (2.65). The longest spinule was found in *T. fascinans* (0.35 mm), and the smallest spinule occurs in *T. mendax* (0.06 mm), *T. bavaricum* (0.05 mm), and *T. skalinskanum* (0.05 mm) (Table 1, Fig. 2, Suppl. material 29: Table S1).

The analysis of the coefficient of variation (CV) indicated that the most variable features were the spinules and the index of achenes. The variation of spinule traits ranged insignificantly from 13.84% (*T. subpolonicum*) to 97.33% (*T. skalinskanum*), and the index ranged from 7.73% (*T. subpolonicum*) to 18.66% (*T. fascinans*). The lowest variation of the coefficient of variation was observed in the length of achenes: it ranged from 2.05% (*T. subpolonicum*) to 12.25% (*T. bellicum*).

**Figure 3.**
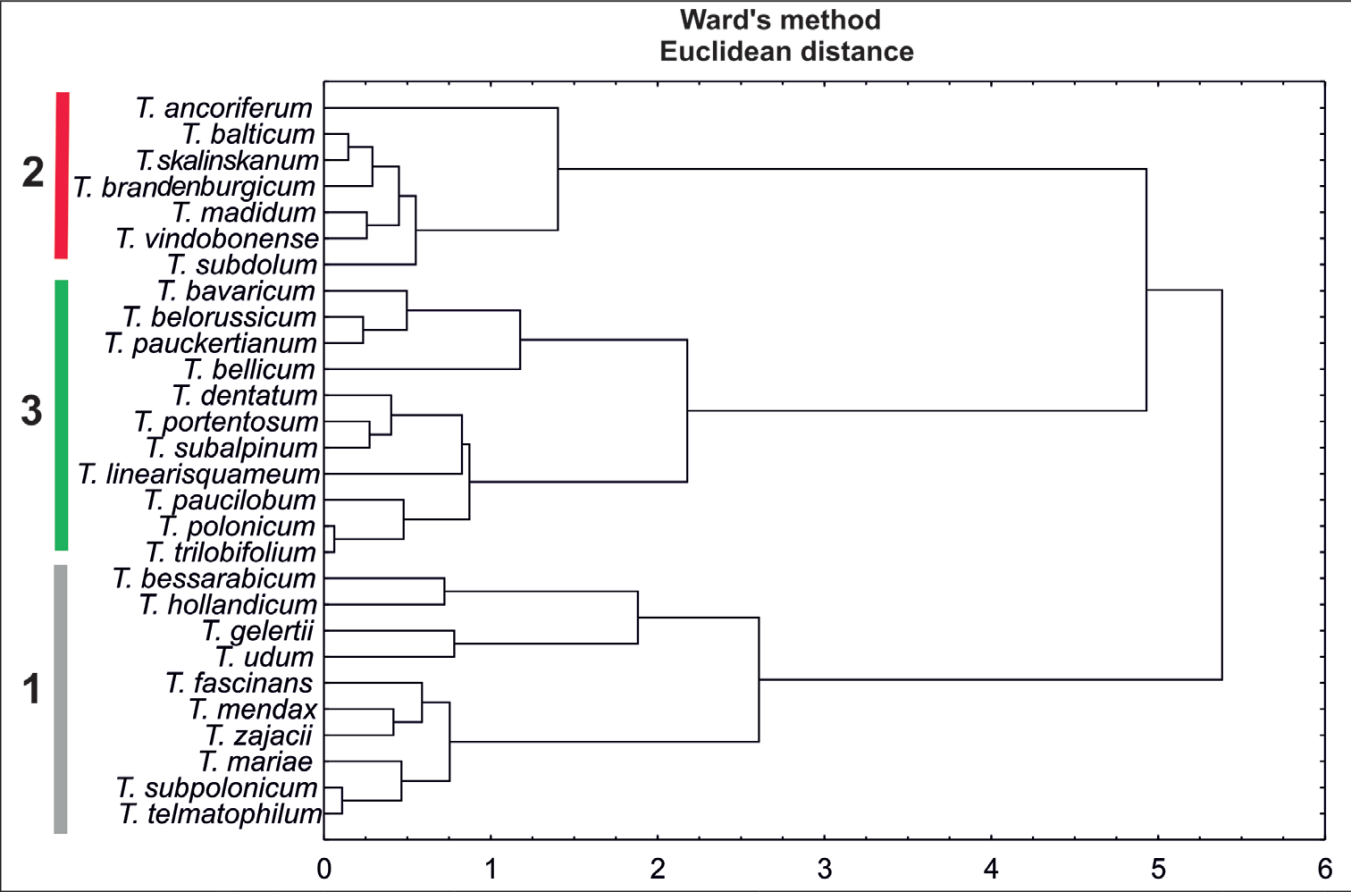
Dendrogram of similarities of *Taraxacum* taxa based on Euclidean distances.

The similarity analysis using Euclidean’s distances showed three main clusters (Fig. 3). The first cluster comprises species with the longest index (*T. hollandicum*, *T. udum*, *T. gelertii*), and species with the widest achenes (*T. telmatophilum*, *T. zajacii*, and *T. mariae*). In the second cluster, most species are characterized by the shortest index and the longest cone (*T. balticum*, *T. skalinskanum*, *T. brandenburgicum*, *T. madidum*, *T. vindobonense*, and *T. subdolum*). In this cluster, the most distantly placed species is *T. ancoriferum* (the species was characterized by the longest and widest achenes and the longest cone). The last cluster is characterized by the shortest achenes and the shortest cone.

**Figure 4.**
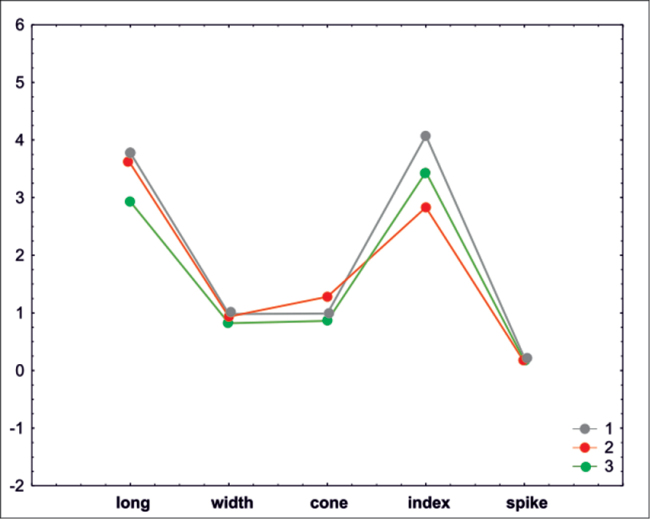
Graph of average clusters determined by K-means clustering analysis for species of *Taraxacum*. Number of K-groups: 1: gray, 2: red, 3: black.

The elements of clusters received by K-means clustering analysis mostly correspond to clusters determined by similarity analysis. The first cluster contains mainly the species with the highest values for the length and the index. The second cluster encompasses species with the longest cone and the lowest index. The third cluster is a group of species with the shortest achenes (Fig. 4, Table 2).

**Figure 5.**
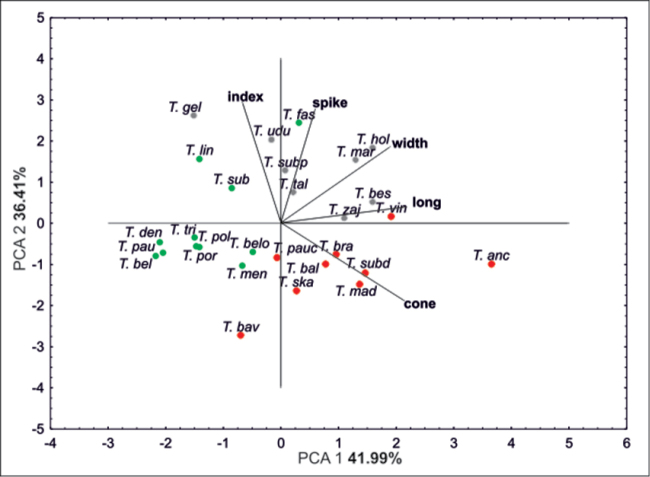
Principal component analysis (PCA) based on five morphological traits.

Ordination diagrams of PCA of *Taraxacum* species based on five morphometric traits revealed that the first two principal components explained 80.66% of the total variance. The first component accounted for 36.41% of the total variance, and the second component accounted for 41.99% of the total variance (Fig. 5). Also, the distribution of species on the PCA diagram confirms the distinctiveness of *T. ancoriferum*. Other species are grouped closely together according to the division obtained on the similarity dendrogram.

**Table 2.** Species of *Taraxacum* belonging to three K-groups; bold: species from the outgroup sections of *Taraxacum*.

Number of K-groups		
1	2	3
***T. bessarabicum***	*T. ancoriferum*	***T. bellicum***
***T. gelertii***	*T. balticum*	*T. belorussicum*
*T. hollandicum*	*T. bavaricum*	*T. dentatum*
*T. mariae*	*T. brandenburgicum*	*T. fascinans*
*T. subpolonicum*	*T. madidum*	***T. linearisquameum***
*T. telmatophilum*	*T. pauckertianum*	*T. mendax*
*T. udum*	*T. skalinskanum*	*T. paucilobum*
*T. zajacii*	*T. subdolum*	*T. polonicum*
	*T. vindobonense*	*T. portentosum*
		*T. subalpinum*
		*T. trilobifolium*

### Ornamentation on the achene body

The ultrastructure of the achene body as revealed by SEM showed a significant variation among species.

The criterion for the division of the analyzed species was the length of spinules. On this basis, three types are distinguished. The first type A) contains species whose surface is covered by very short adjacent spinules tapering towards the end; with a free part of the length measuring from 5 to 10 microns (Suppl. materials 1–9); the second type B) contains species covered with medium length spinules with a free part length from 11 to 19 microns (Suppl. materials 10–22); the last type C) comprises species with a surface covered with very long spinules mainly overlapping with a free part length from 20 to 30 microns (Suppl. materials 23–28). In all groups, tiny granules of various sizes and densities were found on the surface.

In type A), spinules in most species are arranged irregularly on the surface, the exception being the member of the *Taraxacum* section, *T. linearisquameum*, whose spinules are arranged in rows, evenly side by side. In nine species of type B) spinules are spaced apart so as not to overlap; only in three species (*T. trilobifolium*, *T. udum*, *T. zajacii*) there was a clear adhesion and overlap of these structures. Here also two species are notable for spinules arranged evenly in rows in one line (*T. vindobonense*, *T. belorussicum*). In type C), spinules are irregular only in *T. bessarabicum* (sect. Piesis) and protrude from the surface, and in other species spinules clearly adhere to the surface and overlap.

## Diagnostic keys for Taraxacum sect. Palustria fruit from Poland

**Table:** 

1	Large achenes 3.75–5.55 mm with a long cone 1.2–2.0 mm	***T. ancoriferum***
1*	Achenes smaller up to 3.75 mm or a shorter cone	**2**
2	Achenes with a large index, on average more than 4.35 mm	**3**
2*	Achenes with a smaller index	**4**
3	Large achenes 3.85–4.7 mm, very short tight spinules	***T. hollandicum***
3*	Small achenes 3.25–4.1 mm, medium tight spinules	***T. udum***
4	Long and numerous spinules, on average above 0.25 mm	**5**
4*	Spinules shorter and not numerous	**8**
5	Broad achenes 0.9–1.3 mm	***T. fascinans***
6*	Narrower achenes	**7**
7	Large achenes 3.0–3.7 mm	***T. subpolonicum***
7*	Small achenes	***T. subalpinum***
8	Few and very short spinules, below 0.1 mm	**9**
8*	Longer and more numerous spinules	**12**
9	Narrow achenes, on average 0.62 mm, with a short cone (0.85 mm on average)	***T. paucilobum***
9*	Wider achenes with a longer cone	**10**
10	Very short tight spinules	***T. bavaricum***
10*	Medium tight spines	**11**
11	Cone on average 0.95 mm (0.73–1.18)	***T. mendax***
11*	Cone on average 1.2 mm (0.88–1.52)	***T. skalinskanum***
12	Very short tight spinules	**13**
12*	Medium spinules	**15**
12**	Long tight spinules	**20**
13	Small achenes, on average 2.65 mm	***T. dentatum***
13*	Achenes on average above 3.5 mm	**14**
14	Cone on average 1.4 mm, spinules on average 0.2 mm	***T. subdolum***
14*	Cone on average 1.2 mm, spinules on average 0.11 mm	***T. balticum***
15	Spinules arranged in one line	**16**
15*	Spinules irregularly arranged	**17**
16	Broad achenes, on average 1.1 mm	***T. vindobonense***
16*	Achenes narrower, on average 0.85 mm	***T. belorussicum***
17	Cone on average 1.35 mm	***T. madidum***
17*	Shorter cone, on average approx. 1.0 mm	**18**
18	Broad achenes, on average 1.0 mm	***T. zajacii***
18*	Narrow achenes, on average less than 0.8 mm	**19**
19	Achenes on average 3.05 mm long with an index of 3.55	***T. trilobifolium***
19*	Achenes on average 2.9 mm long with an index of 3.2	***T. portentosum***
20	Index less than 3 mm	**21**
20*	Index more than 3.5 mm	**22**
21	Achenes 3.45 mm long, 0.1 mm wide	***T. brandenburgicum***
21*	Achenes on average 3.25 mm long, 0.85 mm wide	***T. pauckertianum***
22	Achenes on average 3.05 mm long, short spinules (on average 0.12 mm)	***T. polonicum***
22*	Achenes above 3.5 mm long, spinules above 0.2 mm	**23**
23	Achenes on average 3.85 mm long, 1.1 mm wide	***T. mariae***
23*	Achenes on average 3.6 mm long, 0.95 mm wide	***T. telmatophilum***

## Discussion

Data provided in the literature on taxonomic studies of Taraxacum sect. Palustria concentrating on achene morphology has mainly provided accurate measurements as in the section monograph (Kirschner and Štěpánek 1998; van Soest 1965) and original species descriptions which are reproduced in subsequent publications (Table 3). None of the articles published so far has provided information on the width of achenes or spinule length. There are also no publications on the micromorphology of marsh dandelion achenes. Our results partly differ from the previous data.

Comparing our research with available literature data is very difficult because of different approaches used by other authors (Table 3). Because of different approaches to measurements, we recorded higher values (length with cone) in five tested species (*T. ancoriferum*, *T. hollandicum*, *T. madidum*, *T. subdolum*, and *T. subpolonicum*) and lower values of three species (*T. portentosum*, *T. bavaricum*, and partly *T. trilobifolium*). The achenes we measured for the other species of marsh dandelions do not differ from the published data, and this also applies to *T. belorussicum*, *T. zajacii*, and *T. mariae*, for which the only information about achene morphology was published in the original species descriptions (Tikhomirov 2003; Marciniuk et al. 2012; Marciniuk et al. 2018). A similar situation occurs with the average cone length; our results for seven analyzed species were higher than the ranges reported in the literature (Table 3). There is a lack of precise biometric data in the literature with which it would be possible to compare the achenes of the species we studied from other sections, i.e., *T. linearisquameum* (sect. Taraxacum), *T. bessarabicum* (sect. Piesis), and *T. gelertii* (sect. Naevosa). The exception is *T. bellicum* (sect. Erythrosperma), from which we obtained results indicating a larger range of achene variation – the achene length (without cone) being 2.02–3.61 mm, the cone length 0.59–1.09 (in our study); compared to research by Dudáš et al. (2013) – achenes 2.3–3.0 mm, cone length 0.8–1.1 mm and the original description of *Taraxacum prunicolor* (Schmid et al. 2004) – achenes 3.8–4.1 mm (with cone), cone length 0.7–1.0 mm. The demonstrated morphometric differences between the achenes studied by other authors and by us may be due to the high plasticity and geographical diversity of the species studied. Previous studies show that the phenotypic plasticity of some *Taraxacum* traits (like leaf size, inflorescence height, or reproductive phenology) is affected by quality and intensity of light (Brock et al. 2005). To answer whether these features are more plastic, detailed research is needed based on material from different locations.

**Table 3.** Comparison of previously published biometric data of achenes of the studied taxa with the outcomes of our research.

	Literature data				This study results			Literature data				This study results		
Species	the achene length (with cone) mm							the cone length						
	Schmid 2002	Kirsch. & Štěp. 1998	Soest 1965	Hudziok 1969	Average	Min	Max	Schmid 2002	Kirsch. & Štěp. 1998	Soest 1965	Hudziok 1969	Average	Min	Max
*T. ancoriferum*	(4.5-)4.7–5.2(-5.5)	(4.5-)4.7–5.2(-5.5)		5.0–5.4	**4.42/6.05**	3.77	5.56	1.2–1.5	1.2–1.5		1.1–1.4	**1.63**	1.22	2.01
*T. balticum*		4.5–5.2(-6.0)			3.55/4.74	3.23	3.98		(0.9-)1.1–1.2(-1.5)			1.19	0.93	1.37
*T. bavaricum*	4.8–5.3	4.8–5.3	4.5		**3.17/4.35**	2.29	3.48	1.6–2.0	1.6–2.0	0.9		1.18	0.94	1.40
*T. belorussicum*					**3.06/4.07**	2.78	3.43					1.01	0.81	1.32
*T. brandenburgicum*		3.8–4.2(-5.0)			3.44/4.65	2.89	4.04		0.8–1.0			**1.21**	0.96	1.63
*T. dentatum*	(2.9-)3.3–3.8	(2.9-)3.3–3.8			2.65/3.42	2.25	3.21	0.5–0.7	0.5–0.7(-0.8)			0.77	0.52	1.05
*T. fascinans*		3.5–4.2			3.12/3.97	2.84	3.47		0.7–1.0			0.85	0.69	1.01
*T. hollandicum*	(4.0-)4.3–4.6(-5.0)	4.3–4.6			**4.30/5.31**	**3.85**	4.70	0.5–0.7(-0.9)	0.5–0.7(-0.9)			**1.01**	0.83	1.35
*T. madidum*	4.0–4.4	4.0–4.4			**3.78/5.12**	3.64	4.14	(0.8-)0.9–1.0(-1.1)	(0.8-)0.9–1.0(-1.1)			**1.34**	1.09	1.53
*T. mariae*					3.87	3.18	4.36					1.03	0.76	1.32
*T. mendax*		(3.8-)4.0–4.2(-4.3)			3.34/4.32	3.02	3.56		(0.8-)0.9–1.1			0.98	0.73	1.18
*T. paucilobum*	4.0–4.5	4.0–4.5			**3.19/4.03**	2.91	3.37	(0.7-)0.8–0.9	(0.7-)0.8–0.9			0.84	0.70	1.01
*T. pauckertianum*	3.8–4.1	3.8–4.1		4.0–4.7	**3.24/4.35**	2.80	3.61	(0.8-)0.9–1.0	0.8-)0.9–1.0		0.5–0.9	**1.11**	0.92	1.45
*T. polonicum*		3.6–4.5			3.05/3.92	2.80	3.30		0.7–0.9			0.87	0.69	1.01
*T. portentosum*		4.1–4.5(-4.8)			**2.93/3.82**	2.71	3.18		0.9–1.2			0.91	0.80	1.06
*T. skalinskanum*		4.8–5.2			3.53/4.74	3.18	3.88		1.2–1.7			1.21	0.88	1.52
*T. subalpinum*	3.7–4.2	3.7–4.2			2.86/3.72	2.65	3.02	0.8–1.0	0.8–0.9			0.86	0.70	1.02
*T. subdolum*		4.2–4.4			**3.53/4.92**	3.15	3.97		1.0–1.4			1.39	1.11	1.63
*T. subpolonicum*		3.5–4.0			**3.53/4.47**	3.01	3.70		0.6–0.8			**0.94**	0.80	1.08
*T. telmatophilum*		4.2–4.5			3.59/4.56	3.02	3.87		0.9–1.0			0.97	0.75	1.13
*T. trilobifolium*	(4.1-)4.2–4.9(-5.1)	(4.1-)4.2–4.9(-5.1)		3.8–4.3	**3.07/3.94**	2.81	3.43	(0.7-)0.8–1.1(-1.2)	(0.7-)0.8–1.1(-1.2)		0.7–1.0	0.87	0.70	1.24
*T. udum*	(4.0-)4.4–4.7(-5.0)	(4.1-)4.4–4.7(-5.1)			3.63/4.45	3.26	4.09	0.5–0.7(-0.9)	0.5–0.9			0.82	0.62	1.08
*T. vindobonense*	(3.8-)4.0–4.3(-5.0)	(3.8-)4.0–4.3(5.0)			3.68/4.94	3.26	3.99	0.7–0.9(-1.0)	0.7–0.9(-1.0)			**1.26**	1.04	1.54
*T. zajacii*					**3.50**	3.00	3.88					1.0	0.78	1.18

The micromorphology analysis of achenes has allowed us to distinguish three main types that do not quite delimit Taraxacum sect. Palustria from other sections. *Taraxacum gelertii* (sect. Naevosa) and *T. bellicum* (sect. Erythrosperma) have the same type of ornamentation as some species from the section Palustria. Our finding is confirmed by previous studies (Savadkoohi et al. 2012; Wu et al. 2011), which showed the lack of proper diagnostic features allowing us to delimit *Taraxacum* sections based on achene micromorphology. In contrast, the features associated with ornamentation are helpful to mark even closely related species. Savadkoohi et al. (2012) presented the results of biometric and ultrastructural studies performed on 17 Asian species belonging to nine sections. Those species were assigned to six groups differing in terms of type of achene ornamentation.

Taraxacum sect. Palustria is a relatively large taxonomic unit with still unknown intra-group relationships. Section monographs (Kirschner and Štěpánek 1998) distinguished geographical and morphological groups of species. The division criteria used – morphological similarity (mainly cage-related features) and similar geographical ranges – are not sufficient to determine actual phylogenetic relationships. However, at least some species within individual groups are probably closely related. The division into groups of species in our research differed in quantitative features of achenes which contrasts to the division of Kirschner and Štěpánek (1998) and the proposals of other authors (Schmid 2002; Uhlemann 2003). In each group, aside from very similar taxa, there were species with clearly different characteristics of leaves and inflorescences. Such incongruence is due to the very complicated reticulate evolution of the genus responsible for the uneven, mosaic distribution of traits (Kirschner et al. 2016). In the first group, species with generally similar morphology are *T. udum* and *T. hollandicum*. Interestingly, the achenes of these species are similar to those of the achenes of taxa from other sections, such as *T. gelertii* (sect. Naevosa), with which they also share a similar leaf shape, and *T. bessarabicum* from the basal section Piesis. Other species of this group, apart from the achenes, differ significantly in the shape and staining of the outer cover leaves and are included in various morphological groups (Kirschner and Štěpánek 1998; Marciniuk et al. 2012, 2018). The second group includes species classified into separate morphological groups by Kirschner and Štěpánek (1998). Nevertheless, *T. ancoriferum*, *T. balticum*, and *T. brandenburgicum* are quite similar in terms of inflorescence morphology. Comparable relations of similarity exist between *T. subdolum* and *T. skalinskanum* as well as *T. vindobonense* and *T. madidum*. The third group includes pairs of species from three taxonomic groups as defined by Kirschner and Štěpánek (1998), namely: 1) *T. bavaricum* and *T. pauckertianum*, 2) *T. paucilobum* and *T. polonicum*, 3) *T. belorussicum* and *T. dentatum*. The other three species: *T. trilobifolium*, *T. portentosum*, and *T. subalpinum*, belong to different morphological groups. *T. subalpinum* is certainly assignable to the *Taraxacum* section; that is further confirmed by the similarity of its achenes to those of *T. linearisquameum* (Taraxacum sect. Taraxacum). *Taraxacum bellicum* from the section Erythrosperma has an isolated position in the group taking into account the color of achenes among all the species tested.

## Conclusion

Achene morphology and micromorphology of *Taraxacum* provide useful diagnostic features. The key presented here may be a useful auxiliary tool (in conjunction with the morphological features of leaves and inflorescences) for the determination of species of Taraxacum sect. Palustria occurring in Poland.

## Supplementary materials

Supplementary material 1 Figure S1 Data type image Explanation note **A** general view of achene of *T. balticum***B–D** achene micromorphology **E, F** sculpture **G** surface 3D ultrastructure. https://binary.pensoft.net/file/469035This dataset is made available under the Open Database License (http://opendatacommons.org/licenses/odbl/1.0/). The Open Database License (ODbL) is a license agreement intended to allow users to freely share, modify, and use this Dataset while maintaining this same freedom for others, provided that the original source and author(s) are credited.Agnieszka Rewicz, Jolanta Marciniuk, Paweł Marciniuk

Supplementary material 2 Figure S2 Data type image Explanation note **A** general view of achene of *T. bavaricum***B–D** achene micromorphology **E, F** sculpture **G** surface 3D ultrastructure. https://binary.pensoft.net/file/469036This dataset is made available under the Open Database License (http://opendatacommons.org/licenses/odbl/1.0/). The Open Database License (ODbL) is a license agreement intended to allow users to freely share, modify, and use this Dataset while maintaining this same freedom for others, provided that the original source and author(s) are credited.Agnieszka Rewicz, Jolanta Marciniuk, Paweł Marciniuk

Supplementary material 3 Figure S3 Data type image Explanation note **A** general view of achene of *T. dentatum***B–D** achene micromorphology **E, F** sculpture **G** surface 3D ultrastructure. https://binary.pensoft.net/file/469037This dataset is made available under the Open Database License (http://opendatacommons.org/licenses/odbl/1.0/). The Open Database License (ODbL) is a license agreement intended to allow users to freely share, modify, and use this Dataset while maintaining this same freedom for others, provided that the original source and author(s) are credited.Agnieszka Rewicz, Jolanta Marciniuk, Paweł Marciniuk

Supplementary material 4 Figure S4 Data type image Explanation note **A** general view of achene of *T. gelertii***B–D** achene micromorphology **E, F** sculpture, **G** surface 3D ultrastructure. https://binary.pensoft.net/file/469038This dataset is made available under the Open Database License (http://opendatacommons.org/licenses/odbl/1.0/). The Open Database License (ODbL) is a license agreement intended to allow users to freely share, modify, and use this Dataset while maintaining this same freedom for others, provided that the original source and author(s) are credited.Agnieszka Rewicz, Jolanta Marciniuk, Paweł Marciniuk

Supplementary material 5 Figure S5 Data type image Explanation note **A** general view of achene of *T. hollandicum***B–D** achene micromorphology **E, F** sculpture **G** surface 3D ultrastructure. https://binary.pensoft.net/file/469039This dataset is made available under the Open Database License (http://opendatacommons.org/licenses/odbl/1.0/). The Open Database License (ODbL) is a license agreement intended to allow users to freely share, modify, and use this Dataset while maintaining this same freedom for others, provided that the original source and author(s) are credited.Agnieszka Rewicz, Jolanta Marciniuk, Paweł Marciniuk

Supplementary material 6 Figure S6 Data type image Explanation note **A** general view of achene of *T. linearisquameum***B–D** achene micromorphology **E, F** sculpture **G** surface 3D ultrastructure. https://binary.pensoft.net/file/469064This dataset is made available under the Open Database License (http://opendatacommons.org/licenses/odbl/1.0/). The Open Database License (ODbL) is a license agreement intended to allow users to freely share, modify, and use this Dataset while maintaining this same freedom for others, provided that the original source and author(s) are credited.Agnieszka Rewicz, Jolanta Marciniuk, Paweł Marciniuk

Supplementary material 7 Figure S7 Data type image Explanation note **A** general view of achene of *T. paucilobum***B–D** achene micromorphology **E, F** sculpture **G** surface 3D ultrastructure. https://binary.pensoft.net/file/469040This dataset is made available under the Open Database License (http://opendatacommons.org/licenses/odbl/1.0/). The Open Database License (ODbL) is a license agreement intended to allow users to freely share, modify, and use this Dataset while maintaining this same freedom for others, provided that the original source and author(s) are credited.Agnieszka Rewicz, Jolanta Marciniuk, Paweł Marciniuk

Supplementary material 8 Figure S8 Data type image Explanation note **A** general view of achene of *T. subdolum***B–D** achene micromorphology **E, F** sculpture **G** surface 3D ultrastructure. https://binary.pensoft.net/file/469041This dataset is made available under the Open Database License (http://opendatacommons.org/licenses/odbl/1.0/). The Open Database License (ODbL) is a license agreement intended to allow users to freely share, modify, and use this Dataset while maintaining this same freedom for others, provided that the original source and author(s) are credited.Agnieszka Rewicz, Jolanta Marciniuk, Paweł Marciniuk

Supplementary material 9 Figure S9 Data type image Explanation note **A** general view of achene of *T. subpolonicum***B–D** achene micromorphology **E, F** sculpture **G** surface 3D ultrastructure. https://binary.pensoft.net/file/469042This dataset is made available under the Open Database License (http://opendatacommons.org/licenses/odbl/1.0/). The Open Database License (ODbL) is a license agreement intended to allow users to freely share, modify, and use this Dataset while maintaining this same freedom for others, provided that the original source and author(s) are credited.Agnieszka Rewicz, Jolanta Marciniuk, Paweł Marciniuk

Supplementary material 10 Figure S10 Data type image Explanation note **A** general view of achene of *T. ancoriferum***B–D** achene micromorphology **E, F** sculpture **G** surface 3D ultrastructure. https://binary.pensoft.net/file/469043This dataset is made available under the Open Database License (http://opendatacommons.org/licenses/odbl/1.0/). The Open Database License (ODbL) is a license agreement intended to allow users to freely share, modify, and use this Dataset while maintaining this same freedom for others, provided that the original source and author(s) are credited.Agnieszka Rewicz, Jolanta Marciniuk, Paweł Marciniuk

Supplementary material 11 Figure S11 Data type image Explanation note **A** general view of achene of *T. bellicum***B–D** achene micromorphology **E, F** sculpture **G** surface 3D ultrastructure. https://binary.pensoft.net/file/469044This dataset is made available under the Open Database License (http://opendatacommons.org/licenses/odbl/1.0/). The Open Database License (ODbL) is a license agreement intended to allow users to freely share, modify, and use this Dataset while maintaining this same freedom for others, provided that the original source and author(s) are credited.Agnieszka Rewicz, Jolanta Marciniuk, Paweł Marciniuk

Supplementary material 12 Figure S12 Data type image Explanation note **A** general view of achene of *T. belorussicum***B–D** achene micromorphology **E, F** sculpture **G** surface 3D ultrastructure. https://binary.pensoft.net/file/469045This dataset is made available under the Open Database License (http://opendatacommons.org/licenses/odbl/1.0/). The Open Database License (ODbL) is a license agreement intended to allow users to freely share, modify, and use this Dataset while maintaining this same freedom for others, provided that the original source and author(s) are credited.Agnieszka Rewicz, Jolanta Marciniuk, Paweł Marciniuk

Supplementary material 13 Figure S13 Data type image Explanation note **A** general view of achene of *T. fascinans***B–D** achene micromorphology **E, F** sculpture **G** surface 3D ultrastructure. https://binary.pensoft.net/file/469046This dataset is made available under the Open Database License (http://opendatacommons.org/licenses/odbl/1.0/). The Open Database License (ODbL) is a license agreement intended to allow users to freely share, modify, and use this Dataset while maintaining this same freedom for others, provided that the original source and author(s) are credited.Agnieszka Rewicz, Jolanta Marciniuk, Paweł Marciniuk

Supplementary material 14 Figure S14 Data type image Explanation note **A** general view of achene of *T. madidum***B–D** achene micromorphology **E, F** sculpture **G** surface 3D ultrastructure. https://binary.pensoft.net/file/469047This dataset is made available under the Open Database License (http://opendatacommons.org/licenses/odbl/1.0/). The Open Database License (ODbL) is a license agreement intended to allow users to freely share, modify, and use this Dataset while maintaining this same freedom for others, provided that the original source and author(s) are credited.Agnieszka Rewicz, Jolanta Marciniuk, Paweł Marciniuk

Supplementary material 15 Figure S15 Data type image Explanation note **A** general view of achene of *T. mendax***B–D** achene micromorphology **E, F** sculpture **G** surface 3D ultrastructure. https://binary.pensoft.net/file/469048This dataset is made available under the Open Database License (http://opendatacommons.org/licenses/odbl/1.0/). The Open Database License (ODbL) is a license agreement intended to allow users to freely share, modify, and use this Dataset while maintaining this same freedom for others, provided that the original source and author(s) are credited.Agnieszka Rewicz, Jolanta Marciniuk, Paweł Marciniuk

Supplementary material 16 Figure S16 Data type image Explanation note **A** general view of achene of *T. portentosum***B–D** achene micromorphology **E, F** sculpture **G** surface 3D ultrastructure. https://binary.pensoft.net/file/469049This dataset is made available under the Open Database License (http://opendatacommons.org/licenses/odbl/1.0/). The Open Database License (ODbL) is a license agreement intended to allow users to freely share, modify, and use this Dataset while maintaining this same freedom for others, provided that the original source and author(s) are credited.Agnieszka Rewicz, Jolanta Marciniuk, Paweł Marciniuk

Supplementary material 17 Figure S17 Data type image Explanation note **A** general view of achene of *T. skalinskanum***B–D** achene micromorphology **E, F** sculpture **G** surface 3D ultrastructure. https://binary.pensoft.net/file/469050This dataset is made available under the Open Database License (http://opendatacommons.org/licenses/odbl/1.0/). The Open Database License (ODbL) is a license agreement intended to allow users to freely share, modify, and use this Dataset while maintaining this same freedom for others, provided that the original source and author(s) are credited.Agnieszka Rewicz, Jolanta Marciniuk, Paweł Marciniuk

Supplementary material 18 Figure S18 Data type image Explanation note **A** general view of achene of *T. subalpinum***B–D** achene micromorphology **E, F** sculpture **G** surface 3D ultrastructure. https://binary.pensoft.net/file/469051This dataset is made available under the Open Database License (http://opendatacommons.org/licenses/odbl/1.0/). The Open Database License (ODbL) is a license agreement intended to allow users to freely share, modify, and use this Dataset while maintaining this same freedom for others, provided that the original source and author(s) are credited.Agnieszka Rewicz, Jolanta Marciniuk, Paweł Marciniuk

Supplementary material 19 Figure S19 Data type image Explanation note **A** general view of achene of *T. trilobifolium***B–D** achene micromorphology **E, F** sculpture **G** surface 3D ultrastructure. https://binary.pensoft.net/file/469052This dataset is made available under the Open Database License (http://opendatacommons.org/licenses/odbl/1.0/). The Open Database License (ODbL) is a license agreement intended to allow users to freely share, modify, and use this Dataset while maintaining this same freedom for others, provided that the original source and author(s) are credited.Agnieszka Rewicz, Jolanta Marciniuk, Paweł Marciniuk

Supplementary material 20 Figure S20 Data type image Explanation note **A** general view of achene of *T. udum***B–D** achene micromorphology **E, F** sculpture **G** surface 3D ultrastructure. https://binary.pensoft.net/file/469053This dataset is made available under the Open Database License (http://opendatacommons.org/licenses/odbl/1.0/). The Open Database License (ODbL) is a license agreement intended to allow users to freely share, modify, and use this Dataset while maintaining this same freedom for others, provided that the original source and author(s) are credited.Agnieszka Rewicz, Jolanta Marciniuk, Paweł Marciniuk

Supplementary material 21 Figure S21 Data type image Explanation note **A** general view of achene of *T. vindobonense***B–D** achene micromorphology **E, F** sculpture **G** surface 3D ultrastructure. https://binary.pensoft.net/file/469054This dataset is made available under the Open Database License (http://opendatacommons.org/licenses/odbl/1.0/). The Open Database License (ODbL) is a license agreement intended to allow users to freely share, modify, and use this Dataset while maintaining this same freedom for others, provided that the original source and author(s) are credited.Agnieszka Rewicz, Jolanta Marciniuk, Paweł Marciniuk

Supplementary material 22 Figure S22 Data type image Explanation note **A** general view of achene of *T. zajacii***B–D** achene micromorphology **E, F** sculpture **G** surface 3D ultrastructure. https://binary.pensoft.net/file/469055This dataset is made available under the Open Database License (http://opendatacommons.org/licenses/odbl/1.0/). The Open Database License (ODbL) is a license agreement intended to allow users to freely share, modify, and use this Dataset while maintaining this same freedom for others, provided that the original source and author(s) are credited.Agnieszka Rewicz, Jolanta Marciniuk, Paweł Marciniuk

Supplementary material 23 Figure S23 Data type image Explanation note **A** general view of achene of *T. bessarabicum***B–D** achene micromorphology **E, F** sculpture **G** surface 3D ultrastructure. https://binary.pensoft.net/file/469056This dataset is made available under the Open Database License (http://opendatacommons.org/licenses/odbl/1.0/). The Open Database License (ODbL) is a license agreement intended to allow users to freely share, modify, and use this Dataset while maintaining this same freedom for others, provided that the original source and author(s) are credited.Agnieszka Rewicz, Jolanta Marciniuk, Paweł Marciniuk

Supplementary material 24 Figure S24 Data type image Explanation note **A** general view of achene of *T. brandenburgicum***B–D** achene micromorphology **E, F** sculpture **G** surface 3D ultrastructure. https://binary.pensoft.net/file/469057This dataset is made available under the Open Database License (http://opendatacommons.org/licenses/odbl/1.0/). The Open Database License (ODbL) is a license agreement intended to allow users to freely share, modify, and use this Dataset while maintaining this same freedom for others, provided that the original source and author(s) are credited.Agnieszka Rewicz, Jolanta Marciniuk, Paweł Marciniuk

Supplementary material 25 Figure S25 Data type image Explanation note **A** general view of achene of *T. mariae***B–D** achene micromorphology **E, F** sculpture, **G** surface 3D ultrastructure. https://binary.pensoft.net/file/469058This dataset is made available under the Open Database License (http://opendatacommons.org/licenses/odbl/1.0/). The Open Database License (ODbL) is a license agreement intended to allow users to freely share, modify, and use this Dataset while maintaining this same freedom for others, provided that the original source and author(s) are credited.Agnieszka Rewicz, Jolanta Marciniuk, Paweł Marciniuk

Supplementary material 26 Figure S26 Data type image Explanation note **A** general view of achene of *T. pauckertianum***B–D** achene micromorphology **E, F** sculpture **G** e surface 3D ultrastructure. https://binary.pensoft.net/file/469059This dataset is made available under the Open Database License (http://opendatacommons.org/licenses/odbl/1.0/). The Open Database License (ODbL) is a license agreement intended to allow users to freely share, modify, and use this Dataset while maintaining this same freedom for others, provided that the original source and author(s) are credited.Agnieszka Rewicz, Jolanta Marciniuk, Paweł Marciniuk

Supplementary material 27 Figure S27 Data type image Explanation note **A** general view of achene of *T. polonicum***B–D** achene micromorphology **E, F** sculpture **G** surface 3D ultrastructure. https://binary.pensoft.net/file/469065This dataset is made available under the Open Database License (http://opendatacommons.org/licenses/odbl/1.0/). The Open Database License (ODbL) is a license agreement intended to allow users to freely share, modify, and use this Dataset while maintaining this same freedom for others, provided that the original source and author(s) are credited.Agnieszka Rewicz, Jolanta Marciniuk, Paweł Marciniuk

Supplementary material 28 Figure S28 Data type image Explanation note **A** general view of achene of *T. telmatophilum***B–D** achene micromorphology **E, F** sculpture **G** surface 3D ultrastructure. https://binary.pensoft.net/file/469060This dataset is made available under the Open Database License (http://opendatacommons.org/licenses/odbl/1.0/). The Open Database License (ODbL) is a license agreement intended to allow users to freely share, modify, and use this Dataset while maintaining this same freedom for others, provided that the original source and author(s) are credited.Agnieszka Rewicz, Jolanta Marciniuk, Paweł Marciniuk

Supplementary material 29 Table S1 Data type image Explanation note **A–E** Biometric characteristics of Taraxacum species: x – arithmetic means, min. max – minimum, maximum, CV – variation coefficients, SD–standard deviation. https://binary.pensoft.net/file/469063This dataset is made available under the Open Database License (http://opendatacommons.org/licenses/odbl/1.0/). The Open Database License (ODbL) is a license agreement intended to allow users to freely share, modify, and use this Dataset while maintaining this same freedom for others, provided that the original source and author(s) are credited.Agnieszka Rewicz, Jolanta Marciniuk, Paweł Marciniuk
