# Supplementary material for: Achene micromorphology and its taxonomic significance in some species in Taraxacum sect. Palustria (Asteraceae)
Source: PhytoKeys. 2020 Oct 29;166:1–28. doi: 10.3897/phytokeys.166.54271 (PMC7661953; doi:10.3897/phytokeys.166.54271)
Supplement: Supplementary material 29 — Table S1 [file phytokeys-166-001-s029.docx]

**Table S1**. A-E. Biometric characteristics of *Taraxacum* species: x - arithmetic means, min. max - minimum, maximum, CV- variation coefficients, SD – standard deviation.

1. Long

| Taxon | **x** | **Min** | **Max** | **SD** | **CV (%)** |
| --- | --- | --- | --- | --- | --- |
| *T. ancoriferum* | **4.42** | 3.77 | 5.56 | 0.45 | 10.14 |
| *T. balticum* | 3.55 | 3.23 | 3.98 | 0.18 | 4.97 |
| *T. bavaricum* | 3.17 | 2.29 | 3.48 | 0.29 | 9.06 |
| *T. bellicum* | 2.34 | 2.02 | 3.61 | 0.29 | 12.25 |
| *T. belorussicum* | 3.06 | 2.78 | 3.43 | 0.16 | 5.22 |
| *T. bessarabicum* | **4.54** | 3.72 | 5.59 | 0.37 | 8.08 |
| *T. brandenburgicum* | 3.44 | 2.89 | 4.04 | 0.33 | 9.54 |
| *T. dentatum* | 2.65 | 2.25 | 3.21 | 0.25 | 9.43 |
| *T. fascinans* | 3.12 | 2.84 | 3.47 | 0.15 | 4.91 |
| *T. gelertii* | 3.28 | 2.87 | 3.55 | 0.16 | 4.97 |
| *T. hollandicum* | **4.30** | 3.85 | 4.70 | 0.20 | 4.74 |
| *T. linearisquameum* | 2.63 | 2.46 | 2.95 | 0.09 | 3.57 |
| *T. madidum* | 3.78 | 3.64 | 4.14 | 0.12 | 3.27 |
| *T. mariae* | 3.87 | 3.18 | 4.36 | 0.21 | 5.46 |
| *T. mendax* | 3.34 | 3.02 | 3.56 | 0.12 | 3.65 |
| *T. paucilobum* | 3.19 | 2.91 | 3.37 | 0.11 | 3.39 |
| *T. pauckertianum* | 3.24 | 2.80 | 3.61 | 0.19 | 5.72 |
| *T. polonicum* | 3.05 | 2.80 | 3.30 | 0.10 | 3.20 |
| *T. portentosum* | 2.93 | 2.71 | 3.18 | 0.10 | 3.43 |
| *T. skalinskianum* | 3.53 | 3.18 | 3.88 | 0.19 | 5.41 |
| *T. subalpinum* | 2.86 | 2.65 | 3.02 | 0.10 | 3.41 |
| *T. subdolum* | 3.53 | 3.15 | 3.97 | 0.13 | 3.76 |
| *T. subpolonicum* | 3.53 | 3.01 | 3.70 | 0.11 | 3.26 |
| *T. telmatophilum* | 3.59 | 3.02 | 3.87 | 0.18 | 5.07 |
| *T. trilobifolium* | 3.07 | 2.81 | 3.43 | 0.13 | 4.30 |
| *T. udum* | 3.63 | 3.26 | 4.09 | 0.15 | 4.24 |
| *T. vindobonense* | 3.68 | 3.26 | 3.99 | 0.14 | 3.90 |
| *T. zajacii* | 3.50 | 3.00 | 3.88 | 0.15 | 4.16 |

1. Width

| Taxon | **x** | **Min** | **Max** | **SD** | **CV (%)** |
| --- | --- | --- | --- | --- | --- |
| *T. ancoriferum* | **1.11** | 0.97 | 1.23 | 0.07 | 6.46 |
| *T. balticum* | 0.97 | 0.84 | 1.21 | 0.10 | 10.65 |
| *T. bavaricum* | 0.69 | 0.51 | 0.83 | 0.09 | 12.92 |
| *T. bellicum* | 0.68 | 0.52 | 0.93 | 0.08 | 12.37 |
| *T. belorussicum* | 0.88 | 0.70 | 1.06 | 0.07 | 7.98 |
| *T. bessarabicum* | 0.84 | 0.73 | 0.96 | 0.05 | 6.49 |
| *T. brandenburgicum* | 0.98 | 0.75 | 1.28 | 0.11 | 11.18 |
| *T. dentatum* | 0.77 | 0.59 | 1.06 | 0.12 | 15.26 |
| *T. fascinans* | **1.11** | 0.90 | 1.30 | 0.11 | 9.82 |
| *T. gelertii* | 0.90 | 0.66 | 1.13 | 0.08 | 9.10 |
| *T. hollandicum* | **1.14** | 0.88 | 1.31 | 0.08 | 7.25 |
| *T. linearisquameum* | 1.02 | 0.80 | 1.22 | 0.10 | 10.11 |
| *T. madidum* | 0.95 | 0.79 | 1.06 | 0.06 | 6.83 |
| *T. mariae* | **1.10** | 0.98 | 1.25 | 0.08 | 7.55 |
| *T. mendax* | 0.85 | 0.61 | 1.04 | 0.08 | 9.36 |
| *T. paucilobum* | 0.62 | 0.41 | 0.78 | 0.08 | 12.91 |
| *T. pauckertianum* | 0.84 | 0.68 | 1.07 | 0.10 | 11.37 |
| *T. polonicum* | 0.76 | 0.61 | 0.95 | 0.09 | 11.34 |
| *T. portentosum* | 0.72 | 0.61 | 0.86 | 0.07 | 10.20 |
| *T. skalinskianum* | 0.83 | 0.17 | 1.03 | 0.14 | 16.33 |
| *T. subalpinum* | 0.90 | 0.73 | 1.02 | 0.07 | 8.13 |
| *T. subdolum* | 0.93 | 0.80 | 1.07 | 0.07 | 7.39 |
| *T. subpolonicum* | 0.92 | 0.80 | 1.04 | 0.06 | 6.34 |
| *T. telmatophilum* | 0.95 | 0.75 | 1.11 | 0.09 | 9.11 |
| *T. trilobifolium* | 0.73 | 0.61 | 0.86 | 0.06 | 8.28 |
| *T. udum* | 0.98 | 0.82 | 1.13 | 0.08 | 8.46 |
| *T. vindobonense* | **1.11** | 0.94 | 1.31 | 0.08 | 7.37 |
| *T. zajacii* | 1.02 | 0.80 | 1.22 | 0.09 | 8.83 |

1. Cone

| Taxon | **x** | **Min** | **Max** | **SD** | **CV (%)** |
| --- | --- | --- | --- | --- | --- |
| *T. ancoriferum* | **1.63** | 1.22 | 2.01 | 0.22 | 13.48 |
| *T. balticum* | 1.19 | 0.93 | 1.37 | 0.10 | 8.65 |
| *T. bavaricum* | 1.18 | 0.94 | 1.40 | 0.12 | 9.93 |
| *T. bellicum* | 0.84 | 0.59 | 1.09 | 0.12 | 14.05 |
| *T. belorussicum* | 1.01 | 0.81 | 1.32 | 0.09 | 9.33 |
| *T. bessarabicum* | 1.22 | 0.96 | 1.48 | 0.13 | 10.82 |
| *T. brandenburgicum* | 1.21 | 0.96 | 1.63 | 0.14 | 11.51 |
| *T. dentatum* | 0.77 | 0.52 | 1.05 | 0.14 | 18.49 |
| *T. fascinans* | 0.85 | 0.69 | 1.01 | 0.08 | 9.95 |
| *T. gelertii* | 0.65 | 0.51 | 0.78 | 0.06 | 9.46 |
| *T. hollandicum* | 1.01 | 0.83 | 1.35 | 0.11 | 11.21 |
| *T. linearisquameum* | 0.68 | 0.57 | 0.81 | 0.06 | 9.49 |
| *T. madidum* | 1.34 | 1.09 | 1.53 | 0.12 | 9.12 |
| *T. mariae* | 1.03 | 0.76 | 1.32 | 0.13 | 12.42 |
| *T. mendax* | 0.98 | 0.73 | 1.18 | 0.10 | 10.25 |
| *T. paucilobum* | 0.84 | 0.70 | 1.01 | 0.07 | 8.88 |
| *T. pauckertianum* | 1.11 | 0.92 | 1.45 | 0.13 | 12.00 |
| *T. polonicum* | 0.87 | 0.69 | 1.01 | 0.08 | 9.01 |
| *T. portentosum* | 0.91 | 0.80 | 1.06 | 0.06 | 6.97 |
| *T. skalinskianum* | 1.21 | 0.88 | 1.52 | 0.18 | 15.17 |
| *T. subalpinum* | 0.86 | 0.70 | 1.02 | 0.10 | 11.14 |
| *T. subdolum* | 1.39 | 1.11 | 1.63 | 0.11 | 7.62 |
| *T. subpolonicum* | 0.94 | 0.80 | 1.08 | 0.07 | 7.53 |
| *T. telmatophilum* | 0.97 | 0.75 | 1.13 | 0.08 | 8.16 |
| *T. trilobifolium* | 0.87 | 0.70 | 1.24 | 0.11 | 12.82 |
| *T. udum* | 0.82 | 0.62 | 1.08 | 0.09 | 11.17 |
| *T. vindobonense* | **1.26** | 1.04 | 1.54 | 0.10 | 8.01 |
| *T. zajacii* | **1.0** | 0.78 | 1.18 | 0.08 | 8.34 |

1. Index

| Taxon | **x** | **Min** | **Max** | **SD** | **CV (%)** |
| --- | --- | --- | --- | --- | --- |
| *T. ancoriferum* | 2.70 | 1.27 | 3.53 | 0.37 | 13.71 |
| *T. balticum* | 3.02 | 2.43 | 4.02 | 0.32 | 10.56 |
| *T. bavaricum* | 2.65 | 1.33 | 3.40 | 0.36 | 13.42 |
| *T. bellicum* | 2.76 | 2.13 | 3.61 | 0.31 | 11.09 |
| *T. belorussicum* | 3.05 | 2.54 | 3.86 | 0.30 | 9.88 |
| *T. bessarabicum* | 3.76 | 2.87 | 4.73 | 0.42 | 11.18 |
| *T. brandenburgicum* | 2.81 | 1.94 | 3.78 | 0.34 | 12.09 |
| *T. dentatum* | 3.49 | 2.52 | 4.80 | 0.47 | 13.55 |
| *T. fascinans* | 3.63 | 0.17 | 4.97 | 0.68 | 18.66 |
| *T. gelertii* | **5.14** | 3.95 | 6.54 | 0.57 | 11.04 |
| *T. hollandicum* | **4.34** | 3.54 | 5.42 | 0.47 | 10.93 |
| *T. linearisquameum* | 3.92 | 3.26 | 5.00 | 0.44 | 11.33 |
| *T. madidum* | 2.86 | 2.38 | 3.60 | 0.30 | 10.58 |
| *T. mariae* | 3.81 | 2.88 | 5.25 | 0.48 | 12.54 |
| *T. mendax* | 3.45 | 2.73 | 4.41 | 0.38 | 11.12 |
| *T. paucilobum* | 3.87 | 3.07 | 4.93 | 0.41 | 10.64 |
| *T. pauckertianum* | 2.94 | 2.41 | 3.45 | 0.24 | 8.31 |
| *T. polonicum* | 3.53 | 3.07 | 4.45 | 0.33 | 9.37 |
| *T. portentosum* | 3.21 | 2.67 | 3.79 | 0.27 | 8.48 |
| *T. skalinskianum* | 3.00 | 2.40 | 3.79 | 0.41 | 13.65 |
| *T. subalpinum* | 3.38 | 2.70 | 4.03 | 0.35 | 10.51 |
| *T. subdolum* | 2.56 | 2.04 | 3.22 | 0.24 | 9.41 |
| *T. subpolonicum* | 3.77 | 3.24 | 4.47 | 0.29 | 7.73 |
| *T. telmatophilum* | 3.72 | 3.11 | 4.72 | 0.37 | 10.01 |
| *T. trilobifolium* | 3.57 | 2.36 | 4.40 | 0.49 | 13.62 |
| *T. udum* | **4.46** | 3.57 | 5.89 | 0.48 | 10.82 |
| *T. vindobonense* | 2.94 | 2.40 | 3.60 | 0.29 | 9.71 |
| *T. zajacii* | 3.59 | 2.92 | 4.81 | 0.38 | 10.53 |

1. Spike

| Taxon | **x** | **Min** | **Max** | **SD** | **CV (%)** |
| --- | --- | --- | --- | --- | --- |
| *T. ancoriferum* | 0.18 | 0.12 | 0.27 | 0.05 | 25.14 |
| *T. balticum* | 0.11 | 0.07 | 0.18 | 0.03 | 28.41 |
| *T. bavaricum* | 0.05 | 0.02 | 0.09 | 0.01 | 30.71 |
| *T. bellicum* | 0.21 | 0.10 | 0.33 | 0.05 | 23.38 |
| *T. belorussicum* | 0.15 | 0.10 | 0.24 | 0.03 | 21.95 |
| *T. bessarabicum* | 0.23 | 0.16 | 0.35 | 0.05 | 20.54 |
| *T. brandenburgicum* | 0.17 | 0.03 | 0.34 | 0.07 | 42.16 |
| *T. dentatum* | 0.11 | 0.06 | 0.23 | 0.04 | 32.16 |
| *T. fascinans* | **0.35** | 0.22 | 0.52 | 0.07 | 18.97 |
| *T. gelertii* | 0.21 | 0.11 | 0.31 | 0.04 | 20.29 |
| *T. hollandicum* | 0.19 | 0.12 | 0.32 | 0.05 | 24.56 |
| *T. linearisquameum* | 0.21 | 0.14 | 0.27 | 0.03 | 15.92 |
| *T. madidum* | 0.11 | 0.04 | 0.20 | 0.04 | 39.68 |
| *T. mariae* | 0.24 | 0.12 | 0.40 | 0.06 | 23.93 |
| *T. mendax* | 0.06 | 0.03 | 0.10 | 0.02 | 31.63 |
| *T. paucilobum* | 0.09 | 0.05 | 0.14 | 0.03 | 28.45 |
| *T. pauckertianum* | 0.17 | 0.08 | 0.27 | 0.05 | 30.29 |
| *T. polonicum* | 0.12 | 0.07 | 0.18 | 0.02 | 19.69 |
| *T. portentosum* | 0.17 | 0.11 | 0.28 | 0.04 | 24.25 |
| *T. skalinskianum* | 0.09 | 0.03 | 0.16 | 0.04 | 39.38 |
| *T. subalpinum* | **0.26** | 0.18 | 0.35 | 0.04 | 16.89 |
| *T. subdolum* | 0.20 | 0.11 | 0.26 | 0.04 | 18.13 |
| *T. subpolonicum* | **0.26** | 0.18 | 0.32 | 0.04 | 13.84 |
| *T. telmatophilum* | 0.20 | 0.11 | 0.31 | 0.04 | 21.89 |
| *T. trilobifolium* | 0.15 | 0.04 | 0.25 | 0.04 | 26.86 |
| *T. udum* | 0.22 | 0.15 | 0.32 | 0.04 | 18.07 |
| *T. vindobonense* | 0.23 | 0.15 | 0.34 | 0.04 | 17.78 |
| *T. zajacii* | 0.18 | 0.11 | 0.29 | 0.04 | 20.61 |
